# Experiences from Sweden on developing evidence-based care for the primary care population

**DOI:** 10.1080/02813432.2025.2461047

**Published:** 2025-02-03

**Authors:** Cecilia Björkelund, Karin Mossberg

**Affiliations:** a School of Public Health and Community Medicine, Institute of Medicine, Sahlgrenska Academy, University of Gothenburg, Gothenburg, Sweden; b Research, Education, Development & Innovation, Primary Health Care, Region Västra Götaland, Gothenburg, Sweden

A national system for knowledge-driven management known as the ‘Knowledge steering organization’ intends to implement updated good clinical practice and practice of ranking for the 23 regions in Sweden. The organization aims to implement best available knowledge in health care *via* ‘person-centered care courses’ as a complementary system to the Swedish National guidelines. Almost all care courses start in primary care and General Practice has been engaged to contribute with knowledge from the primary care context, but hitherto there has been lack of clinical research evidence from primary care to assert the primary care perspective. However, there are now good examples of growing primary care clinical research activities.

In Sweden two parallel systems for implementation of evidence-based care exist. The Swedish National Board of Health and Welfare has been working out national guidelines for most chronic diseases for several years, tasked by the government to draw up national guidelines for good care for patients with severe chronic diseases [1]. The National Board of Health and Welfare makes recommendations on treatments, examinations, working methods and other interventions in health care. The guidelines concern diseases and conditions that affect large numbers of people and thus require significant resources. National guidelines indicate benefits and risks of interventions, as well as prioritize the right interventions for those with the greatest need.

However, the National guidelines are primarily intended for decision-makers concerning the allocation of resources within medical care. The second system, a national system for knowledge-driven management known as the ‘Knowledge steering organization’, intends to implement updated good clinical practice and practice of ranking [2]. This is a partly parallel system to the National guidelines for the 23 regions in Sweden, aiming at implementing best available knowledge in health care, but directing health care professionals rather than decision-makers. During the last years, around 40 standardized, person-centered ‘care courses’ (Danish: pakkeforløb) have been formed by the Knowledge steering organization, for example concerning colorectal cancer, diabetic ulcers, heart failure, and depression [3]. As almost all care courses start in primary care, general practice has been engaged in nearly all work groups together with many different representatives from engaged specialties, all with different – and often reduced – knowledge of the special conditions that are present for primary care patients. This means that, in all ‘courses of care’, primary care and General Practice is engaged to contribute with knowledge from primary care context. The guideline formed by the national Knowledge steering organization is the foundation from which the regions facilitate and work for the best available knowledge to be implemented, prioritized and used, to reach the best possible benefit and reducing the harm for the patients.

However, there are several difficulties integrating the primary care context in the guidelines. In all work groups, General Practice is mostly always under-represented, and decisions are made in majority, which is an obstacle to implement evidence from primary care populations. Knowledge about evidence from primary care context is scarcely known by representatives from secondary care level and the work done by primary care representatives to make basic facts known, such as the importance of accessibility and continuity of care as important factors for early diagnosis and the diagnostic process, is time-consuming and struggling. In primary care, prevalence of multimorbidity is high, and around 40% of the patients over the age of 40 have at least two different diagnoses that interact concerning treatment, medication and prognosis [4,5]. Important facts concerning prevalence, incidence, sensitivity and specificity of tests, positive and negative predictive values, case-finding, step-wise diagnostic process, team based care and the importance of accessibility and continuity of care have to be highlighted. Primary care must manage opportunity costs and prioritizations to provide care for those most needed when the resources fall short. Over-diagnosis has to be considered in a primary care context, when cut-offs for diagnosis criteria are constantly lowered. And it has been obvious for all of us representatives from primary care level, who have been engaged in the person-centered care course work, how important clinical research from the primary care context is to form guidelines that are person-centered and manageable in primary care.

Luckily, there are some good examples to expand clinical research in primary care in Sweden. The National research school of General Practice and the academic departments of General Practice/primary care, contribute to a slowly growing bulk of research educated GPs and primary care health personnel. At the National research school of General Practice, almost 80 dissertations have been produced since the start in 2010 [6]. This means that given organizational and economic prerequisites, all these researchers have a national network and can provide well needed clinical research for primary care and for implementation of updated good clinical practice and practice of ranking [7]. Furthermore, Region Västra Götaland has profiled as a good example where possibility for the primary care centres and for primary care research have been beneficial. Large RCTs with around 20–28 engaged primary care centres at a time, and with support from Research and Development departments, have been launched. Especially during the last years, pragmatic cluster randomized controlled trials have been carried out to answer important questions about best available knowledge to be implemented to reach the best possible benefit for the primary care patients. Performing clinical research in primary context, by researchers active in primary care makes the implementation of the best available knowledge for primary care level possible, and there is some clinical evidence for this [8].

We still have a long way to go. At the moment barely around one percent of costs for clinical research is allocated in primary care research in Sweden. To reach the best available knowledge for the primary care level, clinical research in primary care must grow and expand, and be more stimulated. Research activity at the primary care center must be regarded as something positive for primary care and for health care as a whole. It should be subsidized and counted as a positive achievement that will economically contribute to the primary care centre.
